# National survey of corneal cross-linking (CXL) practice patterns in the United Kingdom during 2019

**DOI:** 10.1038/s41433-022-02365-z

**Published:** 2022-12-20

**Authors:** Sally Hayes, Philip Jaycock, Nicholas Rees, Francisco C. Figueiredo, David P. S. O’Brart, Keith M. Meek

**Affiliations:** 1 Structural Biophysics Research Group, School of Optometry and Vision Sciences, Cardiff University, Maindy Road, Cardiff, CF24 4HQ UK; 2 Nuffield Health Bristol Hospital, 3 Clifton Hill, Clifton, Bristol, BS8 1BN UK; 3 Bristol Medical School, University of Bristol, 5 Tyndall Avenue, Bristol, BS8 1UD UK; 4 Department of Ophthalmology, Royal Victoria Infirmary, Newcastle upon Tyne Hospitals NHS Foundation Trust, Queen Victoria Road, Newcastle upon Tyne, NE1 4LP UK; 5 Bioscience Institute, Faculty of Medical Sciences, Newcastle University, Newcastle upon Tyne, UK; 6 Keratoconus Research Institute, Department of Ophthalmology, St Thomas Hospital, London, SE1 7EH UK

**Keywords:** Therapeutics, Health services, Medical imaging

## Abstract

**Objective:**

To provide an insight into trends in corneal cross-linking (CXL) practice in the UK, including criteria for progression of corneal ectasia, identification of patients for CXL, the CXL procedure itself and post-operative management.

**Methods:**

All ophthalmologist members of the UK Cross-linking (UK-CXL) Consortium were invited to complete an online survey about CXL practice for the year 2019. The data collected was anonymised by site and analysed with descriptive statistics.

**Results:**

Responses were received from 16 individual CXL centres (16/38; 42% response rate) and the data represented ~2,000 CXL procedures performed in the UK in 2019. The commonest indication for CXL was progressive keratoconus. Between centres, there were variations in diagnostic evaluation, patient selection for CXL, the CXL procedure and the pre- and post-operative monitoring of patients.

**Conclusion:**

Consistent with the wide number of CXL treatment techniques described in the published literature world-wide, variations in the monitoring of corneal ectasia, indications for CXL, CXL practice and post-CXL follow-up were found to exist between UK-based CXL centres.

## Introduction

Corneal ectasia describes a group of conditions comprising keratoconus, pellucid marginal degeneration, keratoglobus, and post-excimer laser ectasia. Keratoconus is the most common corneal ectasia and typically affects young people in their late teens and early twenties, with the condition often progressing over a period of approximately 20 years [1]. The rate of keratoconus progression has been reported to be higher in paediatric patients than in adults [2–4]. Advances in corneal tomography and tomography have greatly improved the ability to diagnose keratoconus through the earlier detection of corneal shape changes [5].

Corneal collagen cross-linking (CXL) is a minimally invasive procedure that has been shown to halt the progression of corneal ectasias and prevent further loss of vision [6, 7], and thereby reduce the need for corneal transplantations [8]. The standard CXL treatment, which was first trialled in the late 1990’s and early 2000’s, involves the removal of the corneal epithelium and a 30 min application of a 0.1% riboflavin solution (containing 20% dextran) onto the corneal stroma, followed by a 30 min exposure of the tissue to 365 nm UVA light with a fluence of 3 mW/cm^2^ [9]. In 2013, the National Institute for Health and Care Excellence (NICE) advocated the use of CXL as a safe and effective treatment for the management of keratoconus and corneal ectasia [10]. Over the past two decades, numerous CXL treatment variations have been developed and investigated, including accelerated CXL protocols which use higher intensity UVA (in a continuous or pulsed mode) for shorter periods of time, oxygen-assisted accelerated CXL protocols that aim to increase the availability of oxygen and drive the CXL process, and trans-epithelial CXL protocols which seek to achieve safe and effective CXL outcomes without the need for corneal epithelium removal [11].

In 2013, the UK Cross-linking (UK-CXL) Consortium was established to bring together UK-based ophthalmologists, optometrists, and vision scientists with a shared interest in the collaborative advancement of CXL therapy. The current survey was designed to provide an insight into how ophthalmologist members of the UK-CXL Consortium document progression of patients with corneal ectasia, identify and list patients for CXL, perform the CXL therapy and manage post-operative care. By identifying the current trends in CXL, this paper aims to stimulate discussion about research-led best practices and the development of a code of best practice for CXL. The baseline data gathered in this study will serve as a point of reference from which future changes in policy and CXL practice may be evaluated.

## Materials and methods

In May 2021, an e-mail letter of introduction was sent to invite all ophthalmologist members of the UK-CXL Consortium (51 ophthalmologists spread over 38 centres), to complete a short online survey, via an electronic link. The survey was undertaken using the Jisc Online Survey System (a UK based, GDPR compliant system that meets the ISO/IEC 270001 information security standard). Completion of this survey was entirely voluntary, and a single response was requested from each centre. As 2020 was an unusual year, with significant disruption to normal clinical practice in the UK due to the COVID-19 pandemic, all questions in the survey related to CXL practice in 2019.

The survey questions (available as online Supplementary Material) were divided into five sections. Section 1 focused on the ‘Practice Setting’ and required respondents to identify their primary setting for CXL (NHS or private) and the number of CXL procedures performed in that setting in the year 2019. Section 2, ‘Diagnostic Evaluation and Tools’, examined which corneal imaging devices were routinely used for patients with corneal ectasia, the number of scans performed (per patient per examination) and the use of electronic patient record systems to prospectively record ectasia and CXL. Section 3, ‘Patient Selection’, addressed how patients were identified for CXL and how the progression of patients with corneal ectasia was documented. This section also required respondents to select from a list of indications all those that had been treated with CXL at their centre in 2019 - the list included keratoconus treated at onset, keratoconus with documented progression, pellucid marginal degeneration, post-excimer laser ectasia, infectious keratitis, and an option to specify any other indications. Section 4 focused on ‘CXL treatment approach’ to ascertain the use of epithelium-on or epithelium-off CXL, the method of epithelial removal (for epithelium-off CXL) and the riboflavin solutions and CXL light settings routinely used. Questions were also asked about the use of CXL for the treatment of thin corneas (using a selection of modified CXL parameters) and the incidence of same day bilateral CXL procedures. Section 5, ‘Post-operative and follow-up care’, examined ophthalmologists’ approach to the monitoring of paediatric and adult patients after CXL and the planned length of follow-up prior to discharge. Respondents were invited to contribute free text comments throughout the survey to ensure clarity of responses and/or express opinions. The data was anonymised by site and analysed with descriptive statistics by calculating the percentage of respondents with a response in each category.

This study was reviewed and approved by Cardiff University’s School of Optometry and Vision Sciences Research Ethics Committee (SREC; reference: 1562).

## Results

16 survey responses were received from 16 individual UK sites at which CXL was performed in 2019 (referred to herein as CXL centres). The geographical spread of responses was England 94% (15/16); Wales 6% (1/16); Scotland and Northern Ireland 0% (0/16). The survey response rate, based on the number of CXL centres, was 42% (16/38).

### Practice setting

**Fig. 1:**
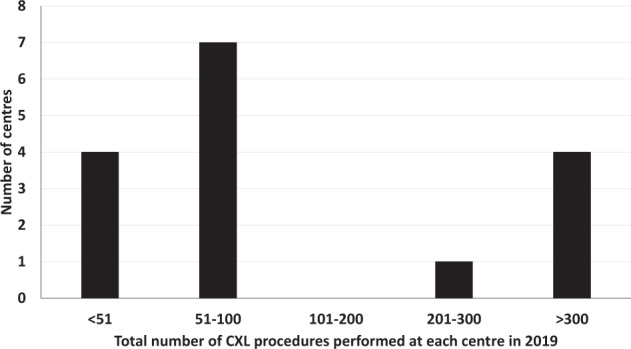
Practice setting. Total number of CXL procedures performed at each centre in 2019.

94% (15/16) reported NHS Hospitals as their primary setting for CXL, with only one respondent (accounting for ≤ 50 of the CXL procedures reported in this survey), citing private practice as their primary setting. Based on a conservative estimate of the data presented in Fig. 1, the survey may be seen to represent ~2000 CXL procedures performed in the UK in 2019.

### Diagnostic evaluation and tools

**Fig. 2:**
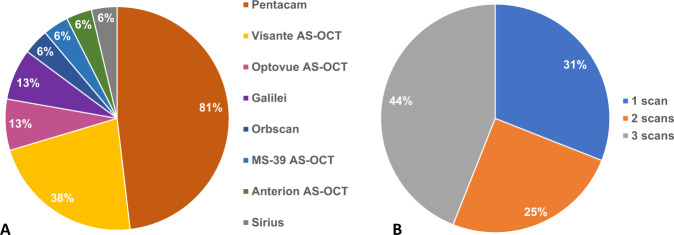
Diagnostic evaluation and tools. **A** Corneal imaging devices routinely used for the monitoring of patients with corneal ectasia (**B**) and the number of scans performed per eye per examination. The corneal imaging devices used included: Pentacam (Oculus, Wetzlar, Germany), Visante anterior segment optical coherence tomography (AS-OCT; Carl Zeiss AG, Jena, Germany), Optovue AS-OCT (Optovue, Fremont, CA), Galilei (Ziemer Ophthalmic Systems AG, Port, Switzerland), Orbscan (Bausch and Lomb, Claremont, CA), MS-39 AS-OCT (CSO, Florence, Italy), Arterion AS-OCT (Heidelberg Engineering, Heidelberg, Germany), Sirius (CSO, Florence, Italy). Results are based on 16 responses from centres performing corneal cross-linking in the UK.

The most used corneal imaging devices for the clinical assessment of patients with corneal ectasia were the Pentacam (Oculus Optikgarate, Germany) and Visante anterior segment Ocular Coherence Tomography (Carl Zeiss, Germany) systems, which were routinely used in 81% (13/16) and 38% (6/16) of the CXL centres, respectively (Fig. 2A). The number of scans routinely performed per eye/per examination (Fig. 2B) was independent of the imaging device used (Fig. 2A). 38% (6/16) routinely performed corneal topography/tomography on the day of CXL. Of the 63% (10/16) that did not perform same-day scanning, most recorded scans within either 1 or 2 months of operating (40% (4/10) and 30% (3/10), respectively).

63% (10/16) of the responding CXL centres confirmed that they did not use an electronic patient record system to prospectively record ectasia in real time. The 38% (6/16) that did prospectively record ectasia in real-time, did so using in-house software (2/16) or bespoke software such as the Keratoconus Module in OpenEyes (Sunderland, UK) (2/16), Medisoft (Leeds, UK) (1/16) or TM3 (Blue Zinc, Belfast, UK) (1/16).

### Patient selection

The most common indications for CXL were progressive keratoconus (100%; 16/16), post-LASIK ectasia (81%; 13/16), and pellucid marginal degeneration (81%; 13/16). 31% (5/16) used CXL for the treatment of infectious keratitis.

Regarding keratoconus, 94% (15/16) did not have a maximal keratometry (Kmax or equivalent) threshold for the routine treatment of keratoconus with CXL. The most common indication for CXL was the documentation of progression by a change in Kmax or equivalent on corneal topography/tomography, with 88% (14/16) reporting the use of Kmax in combination with either a change in K2 and/or a change in visual acuity/refraction to define keratoconus progression. 56% (9/16) reported the use of CXL at presentation (i.e., without waiting for progression) in keratoconus patients aged <18 years old.

Most respondents (88%; 14/16) performed CXL in corneas with a minimum pre-operative thickness (measured at the thinnest point and inclusive of epithelium) of <400 µm. The routine use of CXL in corneas with a thickness of <375 µm or <350 µm was reported by 25% (4/16) and 13% (2/16) of respondents respectively.

### Treatment approach

All respondents (16/16) performed epithelium-off CXL, whilst only 13% (2/16) reported the use of epithelium-on CXL, with the sole indication for epithelium-on CXL being the treatment of thin corneas (<400 µm). For epithelium-off CXL, the method of epithelial debridement was found to be unrelated to the minimum pre-operative thickness of the cornea; 69% (11/16) used ethanol-assisted epithelium removal, 50% (8/16) used manual debridement with a blade or spatula, 13% (2/16) used transepithelial phototherapeutic keratectomy (Trans PTK), and 6% (1/16) performed manual debridement with an Amoils Epithelial Scrubber or similar. 94% (15/16) routinely used an isotonic riboflavin/hydroxypropyl methylcellulose (HPMC) solution for CXL whilst only 6% (1/16) used a riboflavin/dextran solution (Table 1).

**Table 1:** Riboflavin solutions routinely used for epithelium-off corneal cross-linking.

Trade name and manufacturer	Active ingredients	Percentage (and number) of respondents using each formulation*
VibeX Rapid Medio-Haus, Kiel, Germany	0.1% riboflavin, saline, HPMC	81% (13/16)
Mediocross M Medio-Haus, Kiel, Germany	0.1% riboflavin, 1.1% HPMC	13% (2/16)
Mediocross H Medio-Haus, Kiel, Germany^†^	0.1% riboflavin	13% (2/16)
Mediocross D Medio-Haus, Kiel, Germany	0.1 % Riboflavin, 20% Dextran	6% (1/16)
Ribofast Iromed group S.r.l., Rome, Italy	0.1% riboflavin, Vitamin E-TPGS	6% (1/16)

*Data is based on responses from 16 centres performing corneal cross-linking in 2019, with some centres reporting the routine use of more than one type of riboflavin solution.

^†^Mediocross H was used for the treatment of corneas of <400 μm.

All respondents performed accelerated CXL using either a 9 mW (56%; 9/16), 12 mW (6%; 1/16), 18 mW (13%; 2/16) or 30 mW UVA setting (25%; 4/16). Only 31% (5/16) used pulsed UVA. There was no correlation between the minimum pre-operative corneal thickness routinely treated and the UVA intensity/delivery mode employed.

During accelerated CXL, 60% (9/15) routinely rinsed riboflavin from the corneal surface prior to UVA exposure. There was no association between the riboflavin solution used and the decision to rinse or not rinse the cornea before irradiation.

When performing CXL on corneas of <400 µm, 57% (8/14) used a hypotonic riboflavin preparation. Other techniques for the treatment of thin corneas included the use of sterile water (36%; 5/14), contact lens assisted CXL (29%; 4/14), high dose riboflavin (21%; 3/14) and modified UV parameters (7%; 1/14).

63% (10/16) performed same day bilateral CXL in some cases, with an equal division between those that carried out same-day CXL procedures in ≤ 25% of cases and those that performed same-day CXL procedures in > 25% of cases.

### Pre- and post-operative monitoring and follow-up care

Pre-operative and post-operative monitoring results are summarised in Table 2. 19% (3/16) reported the treatment of < 18-year-olds immediately at diagnosis but no one reported treatment at diagnosis for patients aged ≥ 18 years of age. In patients who were monitored prior to undergoing CXL, those aged < 18-years-old were monitored much more frequently than those aged ≥ 18 years of age; 75% (12/16) of respondents reported performing topography/tomography measurements at ≤ 6 monthly intervals for < 18-year-olds whereas only 44% (7/16) reported performing them at ≤ 6 monthly intervals for ≥ 18-year-olds. This trend of monitoring < 18-year-olds more closely was replicated in the post-operative period. In the first post-operative year, most respondents (75%; 12/16) monitored < 18-year-olds at ≤ 6 monthly intervals whereas the monitoring of patients ≥ 18 years of age typically occurred at either 3 to 6 monthly intervals (50%; 8/16) or > 6 monthly intervals (50%; 8/16). Similarly, at > 1 year post-operatively, younger patients (< 18-years-old) were much more likely to be monitored at ≤ 12 monthly intervals than those ≥ 18-years-old. Respondents monitored all CXL patients more closely in the first pre-operative year than thereafter. Most patients were followed up at ≤ 6 monthly intervals in the first year post-operatively compared to ≤ 12 monthly intervals in subsequent years.

**Table 2:** Pre- and post-operative corneal topography/tomography monitoring of paediatric and adult CXL patients.

	Patient age	Corneal topography/tomography interval				
		Treated at diagnosis	≤ 3 months	> 3 to ≤ 6 months	> 6 to ≤ 12 months	> 12 months
**Pre-operative monitoring interval**	< 18 years	19%	31%	44%	6%	N/A
	≥ 18 years	0%	6%	38%	56%	N/A
**1st year post-operative monitoring interval**	< 18 years	N/A	19%	56%	19%	6%
	≥ 18 years	N/A	0%	50%	44%	6%
**>** **1-year post-operative monitoring interval**	< 18 years	N/A	0%	31%	56%	13%
	≥ 18 years	N/A	0%	13%	44%	44%

**Fig. 3:**
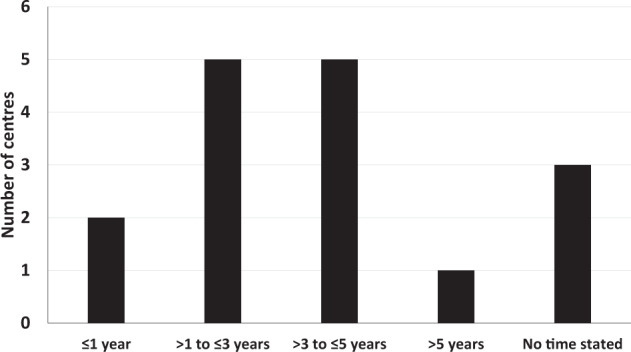
Post-operative follow-up care. Minimum length of time for planned follow-up after corneal cross-linking (based on responses from 16 UK cross-linking centres).

In response to the question: “what is the minimum length of time that you planned to obtain corneal topography/corneal tomography in a hospital eye setting to monitor for progression of ectasia in the treated eye that underwent CXL, before being discharged to a local optometrist?” the planned follow-up times ranged from 9 months to 10 years post-CXL (Fig. 3). A small proportion of respondents (19%; 3/16) did not specify a planned follow-up time, stating that “there was no set minimum length” or that it “depends on the age of the patient and the other eye,” but the majority reported a minimum planned follow-up time of either > 1 to ≤ 3 years (38%, 5/13) or > 3 to ≤ 5 years (38%, 5/13) (Fig. 3).

## Discussion

This survey of CXL practice in 2019 has provided evidence of UK-wide differences in the pre-operative and-post operative monitoring regimens for CXL patients, the indications for CXL, and the CXL treatment technique itself.

Many respondents (63%) did not undertake corneal imaging on the same day as the CXL procedure (likely due to logistical challenges and practical constraints regarding resource allocation), but most recorded scans within 1 month or 2 months pre-operatively. Corneal imaging was most commonly performed using the Scheimpflug Pentacam (Oculus, Wetzlar, Germany) and anterior segment optical coherence tomography Visante system (Carl Zeiss AG, Jena, Germany), which produce very similar measurements of central corneal thickness in healthy and suspected keratoconus patients [12]. However, the irregular surface of the ectatic cornea makes it difficult to achieve good repeatability of corneal topography/tomography [13], especially in cases of advanced keratoconus [14]. The number of corneal topography/tomography scans routinely performed at each examination varied between CXL centres with roughly equal numbers recording 1, 2 or 3 scans. Although time constraints mean that it is not always practicably possible to perform multiple measurements in a clinical setting, the ability to distinguish corneal shape change from measurement error increases significantly when an average of multiple measurements is used, and vast gains in the level of precision can be achieved by increasing the number of measurements from 1 to 3 [15].

Since the advent of CXL, the numerous treatment variations and the lack of consistency in the reporting of CXL outcomes has made it difficult to compare the effectiveness of different protocols. In line with the 2013 NICE recommendations for the audit of CXL clinical outcomes [10], and with the support of the Royal College of Ophthalmologists, the UK-CXL Consortium developed a Keratoconus Module as part of OpenEyes (an open source electronic patient record) for the gathering of specified information from keratoconus patients attending NHS monitoring clinics before and after CXL treatment [16]. However, this survey has shown that in 2019 only 37% of respondents prospectively recorded ectasia and CXL data in the clinic in real time, and use of the Keratoconus Module has been limited by the lack of availability of Open Eyes at many CXL centres.

The predominant indication for CXL was progressive keratoconus, with most documenting progression by a change in Kmax in combination with either a change in K2 and/or a change in visual acuity/refraction. The benefits of corneal tomography and corneal topography systems for the diagnosis and monitoring of ectatic corneal disease are well known in terms of their ability to identify early or subtle changes in corneal thickness and shape [5]. However, the survey results inferred the lack of a standardised definition for keratoconus progression, and the complexity in establishing one that is not technology specific. Although further guidelines are required in this area, a global consensus on keratoconus and ectatic diseases agreed that keratoconus progression should be defined in general terms by a consistent change in two or more of the following parameters: steepening of the anterior or posterior corneal surface; corneal thinning and/or an increase in the rate of corneal thickness change from the periphery to the thinnest point [17].

Literature detailing the pre-operative monitoring of CXL patients is limited and often only referred to briefly in the methodology of published case series. Here it was found that paediatric patients tended to be monitored more closely pre-operatively than adults, mostly being reviewed at 3–6 monthly intervals compared to 6–12 monthly intervals, respectively. This trend was repeated for post-operative follow-up. There are well-established reasons for this more frequent monitoring of paediatric CXL patients: keratoconus tends to progress more unpredictably and at a faster rate in children compared to adults [2–4], paediatric patients typically present at a more severe stage of disease [2], treatment failure occurs more frequently and earlier in children than in adults [6, 7], and poor visual function may have a significant deleterious impact on paediatric personal development [18].

With regards to the CXL treatment itself, riboflavin is critical to both the safety and efficacy of the CXL process, as it enhances cross-link formation whilst also limiting the UVA absorption to the corneal stroma and thereby protecting the inner contents of the eye from damage [9]. Early research into the safety of CXL advised a minimum treatment corneal thickness of 400 µm to keep the radiant exposure of the endothelium, lens and retina within the recommended safety limits for UVA light exposure [19]. However, more recent studies of riboflavin concentration at the endothelium have indicated that it is safe to treat corneas of <400 µm [20]. Indeed, as shown in our survey, 88% of respondents routinely performed CXL on corneas <400 µm, with some operating on corneas <350 µm. Several strategies have been proposed for the safe treatment of thin corneas. Our survey reported that 57% used hypo-osmolar riboflavin solutions to swell the cornea [21]. Less common methods included the use of sterile water to swell the cornea, high concentration riboflavin solutions to increase UVA absorption within the anterior stroma [22], riboflavin-soaked contact lenses (contact-lens assisted CXL) to artificially increase the functional thickness of the cornea [23], and the modification of UV parameters. Since this survey was undertaken, the “sub400” individualised fluence CXL protocol, which uses numerical algorithms based on the thickness of the cornea after riboflavin application to calculate the total UV fluence required, has gained much interest [24]. An early clinical trial of this protocol involving 39 patients with pre-operative corneal thicknesses of <400 µm (with the thinnest being 214 µm) showed corneal stability in 90% of cases at 12-months follow-up [24]. Although larger and more long-term clinical trials are required to validate the safety and efficacy of this protocol it has the potential to expand the eligibility of CXL to an increasing number of keratoconus patients.

In recent years, published CXL studies have shown a notable shift away from the use of riboflavin solutions containing dextran (which can induce corneal thinning) [11], towards those containing HPMC. This preference for riboflavin/HPMC, was also evident in our survey findings and can be attributed to its minimal effect on corneal thickness [11] and its enhanced rate of stromal diffusion which allows the overall treatment time to be shortened [25]. Long-term data directly comparing CXL effectiveness using riboflavin/dextran and riboflavin/HPMC is not yet available. Interestingly, the survey revealed a 60:40 divide between survey respondents that rinsed riboflavin from the corneal surface prior to UVA exposure (during accelerated CXL) and those that left the riboflavin film intact. We are not aware of any published clinical studies examining the benefits/detriment of a corneal rinse prior to UVA. In theory, CXL within the corneal stroma could be enhanced by removing the UVA shielding layer of riboflavin from the corneal surface [26] but conversely, extensive corneal rinsing may lead to wash-out of riboflavin from the anterior-most stroma and a reduction in CXL effectiveness [27]. Clearly the volume and duration of the rinse are important factors in minimising stromal riboflavin washout, but these parameters remain unstandardised and further laboratory studies and prospective clinical trials are required to develop evidence-based guidelines for best practice.

All centres reported the routine use of accelerated CXL, with most (60%) using a UVA intensity of 9 mW and others using higher UVA intensities up to but not exceeding 30 mW. 30 mW is generally considered to be the upper limit for the CXL procedure based on the failure of the Bunsen–Roscoe law of reciprocity at higher UVA intensities [28]. Although accelerated CXL procedures appear to be similarly effective to the standard CXL procedure at halting keratoconus progression [29] and have the advantage of shorter treatment times, their use does not necessarily translate to large scale financial savings, since most of the costs associated with CXL are incurred during the pre- and post-operative monitoring periods rather than during the treatment itself [30].

Bilateral sequential CXL has been reported for small numbers of patients in published studies although outcomes are typically not assessed separately from unilateral cases [7, 31]. This survey shows that in 2019, bilateral CXL was commonly practiced in the UK (63% of responding CXL centres), with 50% of those centres doing so in ≥ 25% of patients. In the most comprehensive study of bilateral CXL practice, Pagano et al. [32] treated 20 keratoconus eyes with sequential bilateral CXL and 18 eyes with unilateral CXL. At 12-months follow-up there was no evidence of progression or complications in the bilateral CXL group but 27% of unilateral CXL patients showed progression of keratoconus in the untreated eye. Economic analysis also showed that four visits were saved for each bilateral treatment compared to unilateral [32]. In patients with relatively fast rates of keratoconus progression or in more complex ectasia cases such as those requiring general anaesthesia, bilateral sequential CXL may therefore be the most suitable and cost-effective option. However, the increase in post-operative pain likely to result from bilateral CXL (which is maximal in the first post-operative 3 days) should be taken into consideration in pre-CXL discussions with the patient and documented in the consent form [33].

Compared to pre-operative monitoring, the regularity of post-operative monitoring of CXL patients tends to be more widely documented in the literature. In a randomised control trial comprising both keratoconus (*n* = 49) and post-LASIK ectasia (*n* = 32) eyes, Hersch et al. [34] found statistically significant changes in Kmax and best corrected distance visual acuity between pre-operative baseline measurements and 1, 3 and 6 months post-operatively but no significant changes in Kmax or best corrected distance visual acuity between 6 months and 12 months [34]. Similarly, in a prospective randomised study of 24 keratoconus eyes with an 18-month follow-up, O’Brart et al. [35] showed that the greatest decrease in Kmean (compared to base-line) occurred at 6 months post-operatively. Whilst others have not demonstrated changes in visual or topographic/tomographic parameters to the level of statistical significance, they too have documented the greatest changes occurring in the first 6 months following CXL [31]. Additionally, CXL complications such as anterior stromal haze and corneal scarring have been reported to frequently diminish over a 12-month follow-up, often with the greatest decrease over the first 6 months [36]. Current UK practice appears to reflect these findings as respondents reported closer monitoring of CXL patients in the first post-operative year (typically ≤ 6 monthly intervals in the first year and ≤ 12 monthly intervals in subsequent years), a system which allows the clinician to assess for evidence of complications and document progress of key visual and topographic/tomographic outcomes during this period of particularly dynamic change. It should be noted however that monitoring CXL patients at 3 monthly intervals as described in some study methodologies would be unfeasible in busy NHS centres with finite resources, particularly after the COVID-19 pandemic.

There are currently no guidelines pertaining to the recommended long-term follow-up of CXL patients, and as highlighted by our survey respondents, the exact duration of planned long-term follow-up after CXL depends on many factors, including the age of the patient, the condition of the other eye and the incidence of eye rubbing. Consistent with this, our survey revealed a wide range of planned follow-up times ranging from 9 months to 10 years post-CXL. Although clinical studies have documented a successful cessation of keratoconus progression in most patients over a period of up to 10-years following use of the standard CXL protocol [6, 7], corneal flattening often continues for several years after CXL [6] and rare reports exist of uncontrolled and continued long-term flattening of corneal curvature beyond 10-years [37]. Such long-term data for accelerated CXL procedures is not yet available but randomised, controlled trials have demonstrated the efficacy of the procedure (using UVA intensities of 9 mW/cm^2^, 18 mW/cm^2^ and 30 mW/cm^2^) at halting keratoconus progression in the short term [29, 38, 39]. At 4-years follow-up, accelerated CXL using 18 mW/cm^2^ UVA (in continuous light mode) for 5 min appears to be similarly effective to the standard CXL protocol in stabilising the cornea [29]. Further randomised controlled trials are required to confirm the long-term efficacy of both continuous-light and pulsed-light, accelerated CXL procedures (especially those that use very high UVA intensities), and to guide recommendations for optimum post-CXL follow-up.

Although this study has successfully fulfilled its purpose of providing an insight into CXL practice in the UK in 2019, it has numerous limitations. The main one is the relatively small number of survey responses (16 in total) and their unequal geographical spread across the UK. The varying response rate from different parts of the UK broadly reflects the geographical distribution of the ophthalmologist members of the UK-CXL Consortium but led to a heavy bias towards CXL performed in NHS centres in England (accounting for 14/16 of the survey responses). It is likely that a proportion of the survey respondents may also have performed CXL in a secondary setting (another hospital or privately) in 2019, using the same or different CXL practices to those reported. Despite these limitations, this baseline survey data nevertheless represents a significant number of CXL procedures performed in the UK in 2019 (~2000 procedures) and the identified variabilities in CXL practices could only be confirmed or increased with a greater number and distribution of responders.

## Summary

### What was known before

- Corneal cross-linking is recognised as a safe and effective treatment for the stabilisation of corneal ectasia.
- Published laboratory and clinical studies have demonstrated the existence of a wide range of corneal cross-linking protocols in use world-wide.

### What this study adds

- This study has identified current trends in corneal cross-linking practice in the UK and highlighted variations between centres in the cross-linking protocol used and the pre- and post-operative monitoring of keratoconus patients.
- As the COVID-19 pandemic has increased demand on hospital services and highlighted the need for optimising patient pathways for monitoring patients with keratoconus and those undergoing corneal cross-linking procedures, this survey stimulates discussion about best practice for cross-linking and the monitoring of keratoconus patients in the UK.

## Supplementary information

Supplementary material_test summary

Supplementary material_survey questions

## Data Availability

The authors confirm that the data supporting the findings of this study are available within the article and its supplementary materials.

## References

[1] Millodot M, Ortenberg I, Lahav-Yacouel K, Behrman S (2016). Effect of ageing on keratoconic corneas. J Optom.

[2] Olivo-Payne A, Abdala-Figuerola A, Hernandez-Bogantes E, Pedro-Aguilar L, Chan E, Godefrooij D (2019). Optimal management of pediatric keratoconus: Challenges and solutions. Clin Ophthalmol.

[3] Léoni-Mesplié S, Mortemousque B, Touboul D, Malet F, Praud D, Mesplié N (2012). Scalability and severity of keratoconus in children. Am J Ophthalmol.

[4] Romano V, Vinciguerra R, Arbabi EM, Hicks N, Rosetta P, Vinciguerra P (2018). Progression of keratoconus in patients while awaiting corneal cross-linking: A prospective clinical study. J Refract Surg.

[5] Belin MW, Villavicencio OF, Ambrósio RR (2014). Tomographic parameters for the detection of keratoconus: Suggestions for screening and treatment parameters. Eye Contact Lens.

[6] O’Brart DP, Patel P, Lascaratos G, Wagh VK, Tam C, Lee J (2015). Corneal Cross-linking to halt the progression of keratoconus and corneal ectasia: Seven-year follow-up. Am J Ophthalmol.

[7] Raiskup F, Theuring A, Pillunat LE, Spoerl E (2015). Corneal collagen crosslinking with riboflavin and ultraviolet-A light in progressive keratoconus: ten-year results. J Cataract Refract Surg.

[8] Godefrooij DA, Gans R, Imhof SM, Wisse RPL (2016). Nationwide reduction in the number of corneal transplantations for keratoconus following the implementation of cross-linking. Acta Ophthalmologica.

[9] Wollensak G, Spoerl E, Seiler T (2003). Riboflavin/Ultraviolet-A-induced collagen crosslinking for the treatment of keratococnus. Am J Ophthalmol.

[10] National Institute for Health and Care Excellence: Photochemical corneal collagen cross‑linkage using riboflavin and ultraviolet A for keratoconus and keratectasia. Interventional procedures guidance [IPG466] www.nice.org.uk/guidance/ipg4662015

[11] Hayes S, Morgan SR, Meek KM (2021). Keratoconus: cross-linking the window of the eye. Therapeutic Adv Rare Dis.

[12] Prospero Ponce CM, Rocha KM, Smith SD, Krueger RR (2009). Central and peripheral corneal thickness measured with optical coherence tomography, Scheimpflug imaging, and ultrasound pachymetry in normal, keratoconus-suspect, and post-laser in situ keratomileusis eyes. J Cataract Refract Surg.

[13] Zheng Y, Huang L, Zhao Y, Wang J, Zheng X, Huang W (2017). Repeatability of corneal elevation maps in keratoconus patients using the tomography matching method. Sci Rep.

[14] Flynn TH, Sharma DP, Bunce C, Wilkins MR (2016). Differential precision of corneal Pentacam HR measurements in early and advanced keratoconus. Br J Ophthalmol.

[15] Brunner M, Czanner G, Vinciguerra R, Romano V, Ahmad S, Batterbury M (2018). Improving precision for detecting change in the shape of the cornea in patients with keratoconus. Sci Rep.

[16] Allan B, Edwards M, Gore D, Kaye S, Kolli S, Meek K, et al. Corneal Crosslinking Data Set: Royal College of Ophthalmologists; 2016 [Available from: https://www.rcophth.ac.uk/wp-content/uploads/2016/08/Corneal-Cross-Linking-Data-Set-July-2016.pdf.

[17] Gomes JA, Tan D, Rapuano CJ, Belin MW, Ambrósio R, Guell JL (2015). Global consensus on keratoconus and ectatic diseases. Cornea.

[18] Kymes SM, Walline JJ, Zadnik K, Sterling J, Gordon MO (2008). Changes in the quality-of-life of people with keratoconus. Am J Ophthalmol.

[19] Spoerl E, Mrochen M, Sliney D, Trokel S, Seiler T. Safety of UVA-riboflavin cross-linking of the cornea. Cornea. 2007;26:385–9. 10.1097/ICO.0b013e3180334f78 17457183

[20] Seiler TG, Batista A, Frueh BE, Koenig K (2019). Riboflavin concentrations at the endothelium during corneal cross-linking in humans. Invest Ophthalmol Vis Sci.

[21] Hafezi F, Mrochen M, Iseli HP, Seiler T (2009). Collagen crosslinking with ultraviolet-A and hypoosmolar riboflavin solution in thin corneas. J Cataract Refract Surg.

[22] Franke MAD, Landes T, Seiler TG, Khayyat D, Johannsmeier S, Heinemann D (2021). Corneal riboflavin gradients and UV-absorption characteristics after topical application of riboflavin in concentrations ranging from 0.1 to 0.5. Exp Eye Res.

[23] Jacob S, Kumar DA, Agarwal A, Basu S, Sinha P, Agarwal A (2014). Contact lens-assisted collagen cross-linking (CACXL): A new technique for cross-linking thin corneas. J Refract Surg.

[24] Hafezi F, Kling S, Gilardoni F, Hafezi N, Hillen M, Abrishamchi R (2021). Individualized corneal cross-linking with riboflavin and UV-A in ultrathin corneas: The Sub400 Protocol. Am J Ophthalmol.

[25] Ehmke T, Seiler TG, Fischinger I, Ripken T, Heisterkamp A, Frueh BE (2016). Comparison of corneal riboflavin gradients using dextran and HPMC solutions. J Refract Surg.

[26] Wollensak G, Aurich H, Wirbelauer C, Sel S (2010). Significance of the riboflavin film in corneal collagen crosslinking. J Cataract Refractive Surg.

[27] Gore DM, O’Brart DP, French P, Dunsby C, Allan BD (2015). A comparison of different corneal iontophoresis protocols for promoting transepithelial riboflavin penetration. Invest Ophthalmol Vis Sci.

[28] Wernli J, Schumacher S, Spoerl E, Mrochen M (2013). The efficacy of corneal cross-linking shows a sudden decrease with very high intensity UV light and short treatment time. Invest Ophthalmol Vis Sci.

[29] Hashemi H, Mohebbi M, Asgari S (2020). Standard and accelerated corneal cross-linking long-term results: A randomized clinical trial. Eur J Ophthalmol.

[30] Godefrooij DA, Mangen M-JJ, Chan E, O’Brart DPS, Imhof SM, de Wit GA (2017). Cost-effectiveness analysis of corneal collagen crosslinking for progressive keratoconus. Ophthalmology.

[31] Hashemi H, Seyedian MA, Miraftab M, Fotouhi A, Asgari S (2013). Corneal collagen cross-linking with riboflavin and ultraviolet a irradiation for keratoconus: Long-term results. Ophthalmology.

[32] Pagano L, Gadhvi KA, Borroni D, Iselin KC, Vinciguerra R, Tzamalis A (2020). Bilateral Keratoconus Progression: Immediate versus delayed sequential bilateral corneal cross-linking. J Refract Surg.

[33] Ghanem VC, Ghanem RC, de Oliveira R (2013). Postoperative pain after corneal collagen cross-linking. Cornea.

[34] Hersh PS, Greenstein SA, Fry KL (2011). Corneal collagen crosslinking for keratoconus and corneal ectasia: One-year results. J Cataract Refract Surg.

[35] O’Brart DP, Chan E, Samaras K, Patel P, Shah SP (2011). A randomised, prospective study to investigate the efficacy of riboflavin/ultraviolet A (370 nm) corneal collagen cross-linkage to halt the progression of keratoconus. Br J Ophthalmol.

[36] Koller T, Mrochen M, Seiler T (2009). Complication and failure rates after corneal crosslinking. J Cataract Refract Surg.

[37] Noor I, Seiler TG, Noor K, Seiler T (2018). Continued long-term flattening after corneal cross-linking for keratoconus. J Refractive Surg.

[38] Gore DM, Leucci MT, Koay SY, Kopsachilis N, Nicolae MN, Malandrakis MI, et al. Accelerated pulsed high-fluence corneal cross-linking for progressive keratoconus. Am J Ophthalmol. 2021;221:9–16. 10.1016/j.ajo.2020.08.021 32818448

[39] Shetty R, Pahuja NK, Nuijts RM, Ajani A, Jayadev C, Sharma C (2015). Current protocols of corneal collagen cross-linking: Visual, refractive, and tomographic outcomes. Am J Ophthalmol.

