# Supplementary material for: National survey of corneal cross-linking (CXL) practice patterns in the United Kingdom during 2019
Source: Eye (Lond). 2022 Dec 20;37(12):2511–7. doi: 10.1038/s41433-022-02365-z (PMC9767393; doi:10.1038/s41433-022-02365-z)
Supplement: Supplementary file 1 — Supplementary material_test summary [file 41433_2022_2365_MOESM1_ESM.docx]

**National survey of corneal cross-linking (CXL) practice patterns in the United Kingdom during 2019**

Sally Hayes [1], Philip Jaycock [2], Nicholas Rees [3], Francisco C. Figueiredo [4,5], David P.S. O’Brart [1,6], Keith M. Meek [1]

**Supplementary material:**

**1. Survey questions**

In May 2021, all ophthalmologist members of the UK Cross-linking (UK-CXL) Consortium were invited to complete a short online survey. The survey was undertaken using the Jisc Online Survey System. The survey questions are available here in the supplementary material file ‘Suppmentary material_Survey questions.pdf’.
