# Supplementary material for: National survey of corneal cross-linking (CXL) practice patterns in the United Kingdom during 2019
Source: Eye (Lond). 2022 Dec 20;37(12):2511–7. doi: 10.1038/s41433-022-02365-z (PMC9767393; doi:10.1038/s41433-022-02365-z)
Supplement: Supplementary file 2 — Supplementary material_survey questions [file 41433_2022_2365_MOESM2_ESM.pdf]

# Survey of UK corneal cross-linking (CXL) practice

## Page 1: About this survey

Dear UK-CXL Consortium members,

We are conducting a baseline survey of corneal cross-linking (CXL) practice patterns in the UK to help answer some of the questions posed by our consortium members and identify relevant topics for discussion at future UK-CXL Consortium meetings. We would be grateful if you could please review the information below and complete the following survey.

**Background information**

The aim of the project is to gain insights into current trends in how ophthalmologists: (i) document progression of patients with corneal ectasia, (ii) identify patients for CXL treatment and (iii) manage post-operative care. In this context, corneal ectasia encompasses keratoconus, pellucid marginal degeneration and post-excimer ectasia (ectasia following laser eye surgery). As 2020 was an unusual year in terms of the number of procedures performed, all questions in this survey relate to CXL practice in the year 2019.

**Privacy and Confidentiality**

The data collected will be anonymised by site and analysed with descriptive statistics. All responses are confidential and data will only be shared in aggregate form in any reports or academic publications that arise from this survey.

**Data Protection**

For the purposes of this survey Cardiff University is the data controller. All data collected in this survey will be held securely by the survey software provider (Jisc) under contract and then retained by Cardiff University, in accordance with the Data Protection Act (1998) for a period of 3 years following the completion of the survey.

**Risks and Benefits of Participation**

There are no immediate or expected risks from participating in this study. It is our intention to publish the results of this survey as an open access publication, in which the contribution of the UK-CXL Consortium will be acknowledged as a whole. The survey will also be used to identify relevant topics for discussion at future UK-CXL Consortium meetings.

**Voluntary nature of the survey**

Completion of this survey is entirely voluntary. If you decide to participate, completion of the survey should take about 10-15 minutes. You may withdraw your responses at any time and for any reason.

**Further queries**

For any further queries relating to this survey, please contact Dr Sally Hayes (Hayess5@cardiff.ac.uk).

**Consent**

By completing this survey, I consent to my name, e-mail address and submitted responses being kept on a database for a period of 3 years, for the purpose of data analysis and to provide the opportunity to go back to individuals to ask further questions if required. The survey will not collect any patient identifiable information.

1. Please type your name to verify your consent.

# Page 2

## Section 1: Practice setting

2. Please indicate the primary setting where you performed CXL in 2019.

- NHS Hospital
- University
- Private Practice
- Other

2.a. Please specify your organisation:

2.b. Please specify your organisation

2.c. Please specify your organisation:

2.d. Please specify your organisation:

3. What was the total number of CXL procedures performed by all staff at your centre during the year 2019?

- ≤50
- 51-100
- 101-200
- 201-300
- >300

## Section 2: Diagnostic evaluation and tools. The following questions relate to routine practices in 2019.

*4.* Which of the following corneal imaging devices did you routinely use in patients with corneal ectasia? Please select all that apply.

- ☐ Pentacam
- ☐ Sirius
- ☐ Galilei
- ☐ Orbscan
- ☐ Visante anterior segment OCT
- ☐ Optovue anterior segment OCT
- ☐ SL-OCT (Heidelberg Engineering)
- ☐ MS-39 anterior segment OCT
- ☐ Other

*4.a.* Please specify:

*5.* Was corneal topography/corneal tomography routinely performed on the day of CXL?

- No

- Yes

5.a. Please specify how many months pre-operatively corneal topography/corneal tomography was routinely measured?

[ ]

6. When corneal topography/corneal tomography was performed, how many scans were routinely performed per eye per examination?

- One scan per examination
- Two scans per examination
- Three scans per examination
- More than three scans per examination

7. Did you use an electronic patient record system to prospectively record ectasia and CXL data in the clinic in real time?

- No
- Yes

7.a. What record system did you use?

- Keratoconus module in OpenEyes (developed by the UK-CXL Consortium)
- Other

7.a.i. Please specify:

[ ]

8. With regards to **Section 2 (Diagnostic evaluation and tools)**, is there any further information that you would like to provide? *Optional*

## Section 3: Patient selection. The following questions relate to your routine practices in 2019.

9. Did you perform CXL for the following indications? Please select all that apply.

- ☐ Progressive keratoconus, with documentation of progression by change in KMax or equivalent on corneal topography/tomography
- ☐ Progressive keratoconus, with documentation of progression by change in K2 or equivalent on corneal topography/tomography
- ☐ Progressive keratoconus, with documentation of progression by a change in refraction or visual acuity
- ☐ Keratoconus at presentation (ie. without waiting for progression) in patients <18 years of age
- ☐ Post-LASIK or Post-PRK ectasia
- ☐ Pellucid marginal degeneration
- ☐ Recurrence of ectasia in corneal grafts
- ☐ Infectious keratitis
- ☐ Other

9.a. Please specify:

10. Was there a maximal keratometry (KMax or equivalent) threshold routinely treated with CXL?

- No
- Yes

10.a. Please specify the maximal keratometry threshold for routine treatment:

11. What was the minimum pre-operative total corneal thickness (measured at the thinnest point and inclusive of epithelium) on corneal topography/corneal tomography that was routinely treated with CXL at your unit?

- < 350 μm
- < 375 μm
- < 400 μm
- ≥ 400 μm

12. With regards to **Section 3 (Patient selection)**, is there any further information that you would like to provide? *Optional*

## Section 4: CXL treatment approach. The following questions relate to your routine practices in 2019.

13. Did you perform epithelium-off CXL?

- ○ No
- ○ Yes

13.a. What method was most frequently used for epithelial removal?

- ☐ Manual (blade/spatula)
- ☐ Manual (EBK device or similar)
- ☐ Manual (Amoils Epithelial Scrubber or similar)
- ☐ Ethanol (Alcohol) assisted
- ☐ Trans PTK
- ☐ Trans PRK
- ☐ Other

13.a.i. Please specify:

14. Which riboflavin preparation(s) did you routinely use for epithelium-off CXL procedures? Please select all that apply.

- ☐ Mediocross D
- ☐ Mediocross M
- ☐ VibeX Rapid
- ☐ Other

14.a. If you selected Other, please specify:

15. Did you perform epithelium-on CXL?

- ○ No
- ○ Yes

15.a. When did you perform epithelium-on CXL

- ☐ Routinely
- ☐ In paediatric patients
- ☐ In patients with thin corneas
- ☐ Other

15.a.i. Please specify:

16. Did you routinely perform accelerated CXL for either epithelium-on or epithelium-off procedures? If so, which of the following UVA settings did you use? Please select all that apply.

- ☐ No
- ☐ Yes, 9 mW
- ☐ Yes, 18 mW
- ☐ Yes, 30 mW
- ☐ Yes, 45 mW
- ☐ Other

16.a. Please specify:

16.b. When performing accelerated CXL procedures did you routinely rinse the riboflavin from the corneal surface prior to UVA exposure?

- ○ No
- ○ Yes

17. Did you routinely perform pulsed CXL for either epithelium-on or epithelium-off procedures?

- ○ No
- ○ Yes

18. Did you routinely treat corneas with a corneal thickness <400 microns? If so, which of the following techniques did you use? Please select all that apply.

- ☐ No
- ☐ Yes, hypotonic riboflavin formulation
- ☐ Yes, sterile water
- ☐ Yes, contact lens assisted
- ☐ Yes, stromal lenticule assisted
- ☐ Yes, high dose riboflavin
- ☐ Yes, modified UV parameters
- ☐ Other

18.a. Please specify:

19. Did you perform same day bilateral CXL in any cases?

- No
- Yes ≤ 25% of cases
- Yes > 25% of cases

20. With regards to **Section 4 (CXL treatment approach)**, is there any further information that you would like to provide? *Optional*

## Section 5: Post-operative and follow-up care. The following questions relate to your routine practices in 2019.

21. At what monthly intervals did you most commonly obtain corneal topography/corneal tomography to monitor for progression of ectasia prior to CXL in patients <18 years of age?

- ≤ 3 months
- > 3 to ≤ 6 months
- > 6 to ≤ 12 months
- Treated at diagnosis

22. At what monthly intervals did you most commonly obtain corneal topography/corneal tomography to monitor for progression of ectasia prior to CXL in patients ≥18 years of age?

- ≤ 3 months
- > 3 to ≤ 6 months
- > 6 to ≤ 12 months
- Treated at diagnosis

23. At what monthly intervals did you most commonly obtain corneal topography/corneal tomography to monitor for progression of ectasia in the treated eye in the first year after CXL in patients <18 years of age?

- ≤ 3 months post-op
- > 3 to ≤ 6 months post-op
- > 6 to ≤ 12 months post-op
- >12 months post-op
- Not applicable, returned to referring provider

24. At what monthly intervals did you most commonly obtain corneal topography/corneal tomography to monitor for progression of ectasia in the treated eye in the first year after CXL in patients ≥18 years of age?

- ≤ 3 months post-op
- > 3 to ≤ 6 months post-op
- > 6 to ≤ 12 months post-op
- >12 months post-op
- Not applicable, returned to referring provider

25. At what monthly intervals did you most commonly obtain corneal topography/corneal tomography to monitor for progression of ectasia in patients in the treated eye that underwent

CXL more than 1 year ago in patients <18 years of age?

- ≤ 3 months post-op
- > 3 to ≤ 6 months post-op
- > 6 to ≤ 12 months post-op
- >12 months post-op
- Not applicable, returned to referring provider

*26.* At what monthly intervals did you most commonly obtain corneal topography/corneal tomography to monitor for progression of ectasia in patients in the treated eye that underwent CXL more than 1 year ago in patients ≥18 years of age?

- ≤ 3 months post-op
- > 3 to ≤ 6 months post-op
- > 6 to ≤ 12 months post-op
- >12 months post-op
- Not applicable, returned to referring provider

*27.* Following CXL, what is the minimum length of time that you planned to obtain corneal topography/corneal tomography in a hospital eye setting to monitor for progression of ectasia in the treated eye that underwent CXL, before being discharged to a local optometrist?

*28.* With regards to **Section 5 (Post-operative and follow-up care)**, is there any further information that you would like to provide? *Optional*

## Further information

29. Can we contact you for additional information? ✱ *Required*

- No
- Yes

29.a. Please provide a contact email address.

## Page 3: Thank you for completing this survey
